# A 7T MRI Study of Fibular Bone Thickness and Density: Impact of Age, Sex and Body Weight, and Correlation with Bone Marrow Expansion and Muscle Fat Infiltration

**DOI:** 10.3390/diagnostics15050564

**Published:** 2025-02-26

**Authors:** Talon Johnson, Jianzhong Su, Anke Henning, Jimin Ren

**Affiliations:** 1 Advanced Imaging Research Center, University of Texas Southwestern Medical Center, Dallas, TX 75390, USA; 2 Department of Mathematics, University of Texas at Arlington, Arlington, TX 76019, USA; 3 Department of Radiology, University of Texas Southwestern Medical Center, Dallas, TX 75390, USA

**Keywords:** fibula, bone, bone marrow, skeletal muscle, fat, aging, osteoporosis, sarcopenia, sex

## Abstract

**Background:** Reduced bone mass and density, hallmark features of osteopenia and osteoporosis, significantly increase the risk of fractures, falls, and loss of mobility, especially in post-menopausal women and the elderly. **Methods:** This quantitative 7T MRI study examines the features of fibular bone thinning and bone mineral density loss (BMD) in 107 individuals (43F/64M) across various ages, body mass indices (BMIs), and ethnicities. **Results:** Women had significantly lower cross-sectional bone wall thickness (BT) and bone tissue area (BA), along with greater BMD loss compared to men in those over age 50 (*n* = 77), but not in the younger group (*n* = 30). The bone g-factor, defined as the ratio of inner-to-outer bone diameters, increased with bone thinning, bone marrow expansion (BME), and muscle fat infiltration (MFI) but was independent of subcutaneous fat thickness (SFT). Bone thinning and BMD loss both tend to increase with BME and MFI. Additionally, bone density decrease correlated with bone mass loss, with a stronger association observed with BT than BA. **Conclusions:** These findings offer insights into the effects of aging and sex on skeletomuscular health, with implications for strategies to mitigate bone loss in osteoporosis and osteosarcopenia.

## 1. Introduction

Bone is a metabolically active tissue that undergoes continuous remodeling throughout life [1]. This dynamic process is highly coordinated, allowing bones to change and reshape in maintaining their strength and integrity while adapting to mechanical stress, repairing damage, redistributing bone mass, and supporting mineral homeostasis [2].

Bone remodeling occurs in a repetitive cycle of activation, resorption, and formation. During the activation phase, specific signals—such as mechanical stress or hormonal changes—activate osteoclasts. Activated osteoclasts resorb (break down) old or damaged bone, creating small cavities, also known as ‘resorption pits, holes, and trails’ [3]. During this phase, calcium (Ca^2+^) and phosphate (HPO_4_^2−^) are released into the bloodstream, contributing to the body’s mineral homeostasis. After the resorption phase, osteoclasts undergo apoptosis (programmed cell death), and osteoblasts are recruited to the resorption cavities. Osteoblasts then lay down a new extracellular matrix, primarily composed of collagen. Initially unmineralized, this new bone gradually mineralizes as hydroxyapatite crystals are deposited, forming a stronger and more resilient bone matrix [4]. While bone remodeling is a vital and necessary process, imbalances—such as increased resorption or reduced formation due to factors like repeated injury, mineral deficiency, and others—can result in net bone mass loss, a condition strongly linked to osteoporosis and osteopenia [5], which affect 200 million people worldwide [6]. As bone mass and density decrease, bones become weaker and more fragile, unable to withstand normal stress. This vulnerability increases the risk of deformities and fractures, leading to chronic pain, reduced mobility, and a significant decline in quality of life [5]. In the U.S., 2 million fragility fractures occur annually, costing more than USD 17 billion in direct annual care. The mortality rate one year after hip fracture is as high as 20–24%, highlighting the importance of managing bone loss [7,8].

Bone loss (BL) is intricately linked to muscle fat infiltration (MFI) and bone marrow expansion (BME) [9,10]. MFI, the excessive accumulation of fat within skeletal muscle, is strongly associated with sarcopenia (progressive loss of muscle mass and function), osteopenia, and osteoporosis. Osteosarcopenia, a condition characterized by concurrent reductions in both muscle and bone mass, is more severe and harder to treat than either condition alone [11,12]. Our recent study has highlighted a strong association between MFI and BME, with both increasing as a result of aging [13]. While aging is a key driver of BL, MFI, and BME, currently, there has been a lack of systematic studies that rigorously link all these measurements from the calf muscle–fibula region using non-invasive imaging techniques. This knowledge gap limits our ability to gain a deeper understanding of, and effectively treat, skeletomuscular conditions that mostly affect daily activities such as walking, standing, jogging, bending, climbing stairs, and getting in and out of chairs.

Magnetic Resonance Imaging (MRI) is a non-invasive modality capable of offering detailed insights into bone and soft tissue structures [13,14]. Additionally, it is easy to integrate MRI with Magnetic Resonance Spectroscopy (MRS) to obtain both structural and metabolic information in a single scan session, thus offering great potential for a more comprehensive understanding of tissue composition and function [15]. T2-weighted (T2w) MRI is particularly effective for assessing fibula bone, as it can clearly distinguish between bone, marrow, and muscle. In cross-sectional T2w MRI, a healthy fibula is typically ring shaped and distinctively hypointense due to the low levels of intraosseous ^1^H signals from water, lipids, and collagen. However, as bone tissue undergoes resorption, water and lipid molecules may infiltrate into the intraosseous cavities, tunnels, and pore spaces excavated by osteoclasts, leading to a rise in bone image intensity. Such image alteration can serve as a valuable imaging marker for assessing bone wall thinning, bone mineral density (BMD) loss, and other morphologic changes.

The current 7T MRI study as an extension of our previous work on muscle fat infiltration (MFI) and bone marrow expansion (BME) will examine the impact of age, sex, and body mass index (BMI) on fibula bone loss, including bone thinning and BMD loss. Compared to conventional MRI, ultrahigh-field MRI provides enhanced T2/T1 and magnetic susceptibility effects, resulting in sharper image contrast that better delineates bone, muscle, and marrow. This facilitates precise segmentation of the fibula bone and accurate measurement of key anatomical parameters, including cross-sectional bone tissue area (BA) and bone wall thickness (BT).

To clarify, throughout this paper, the term ‘bone thinning’ specifically refers to the decrease in bone wall thickness (BT), rather than the outer diameter of the bone shaft. Additionally, the terms ‘bone-wall thickness’ and ‘bone thickness’ are used interchangeably, as are the terms ‘bone-tissue area’ and ‘bone area’. Furthermore, to aid in understanding the bone’s cross-sectional morphological features, we introduce a new term, ‘g-factor’ or ‘g-ratio’, which is defined as the ratio of the bone’s inner-to-outer diameters. This new parameter will be used, as a supplement to BT, to index the bone’s morphological thinning with respect to the width of the enclosed bone marrow tissue. By analyzing these bone-related parameters and their correlations with muscle fat infiltration (MFI) and bone marrow expansion (BME), this study aims to shed more light on the mechanisms of bone thinning and BMD loss, which could inform effective strategies to prevent and treat conditions such as osteoporosis, osteopenia, and sarcopenia.

## 2. Materials and Methods

### 2.1. Subjects and Data Acquisition

MRI images were acquired at 7 Tesla from 107 subjects, comprising 43 females aged 52.7 ± 14.6 years (in the range 15–78 years) and 64 males aged 57.9 ± 17.8 years (in the range 11–79 years). The average BMI was 28.4 ± 4.4 kg/m^2^ (ranged between 19.0 and 38.5) for males and 30.3 ± 5.7 kg/m^2^ (ranged between 19.7 and 44.3) for females. The MRI scan protocol was approved by the Institutional Review the University of Texas Southwestern Medical Center Board and informed consent was obtained from all participants prior to the scan.

All subjects were positioned feet first and supine in the MRI scanner (7T Achieva, Philips Healthcare, Best, The Netherlands), with the calf muscle positioned parallel to the magnetic field and directly on the detection coil (Philips Healthcare). The coil was a partial-volume, double-tuned 1H/31P quadrature coil. The center of the coil was positioned approximately one-third of the distance along the leg from the knee to the heel. Nine slices of axial T2-weighted turbo spin echo images were acquired. Typical parameters were field of view 180 × 180 mm, in-plane spatial resolution 0.7 × 0.7 mm^2^, slice thickness 4 mm, gap 2 mm; repetition time (TR) 2 s, echo time (TE) 75 ms, turbo factor 16, number of acquisitions (NA) = one, and acquisition time 1.5 min.

### 2.2. Data Processing and Analysis

**Figure 1:**
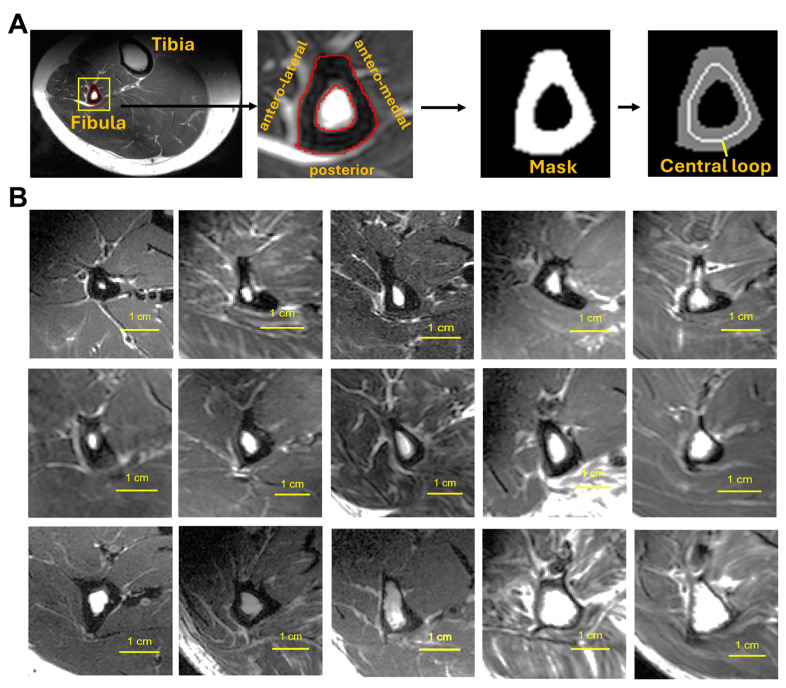
(**A**) Cross-sectional T2-weighted MRI showing the segmentation of the fibula bone for the extraction of bone area (BA) and the length of the central loop (CL). (**B**) A set of 15 randomly selected MR images from the cohort (*n* = 107) illustrating variation in fibula bone size and shape. Note that the fibula bone area exhibits a deformed ring-shaped appearance, with a circular inner contour surrounding the bone marrow and a triangular outer contour bordered by different muscle groups. The triangle points approximately in the anterior direction, with the posterior side bordered by the flexor hallucis longus, the antero-medial side bordered by the tibia posterior, and the antero-lateral side bordered by the peroneus longus.

MRI image processing was performed using the freely available Multi-Image Analysis GUI (Mango, Version 4.1) (https://mangoviewer.com/). Mango’s ROI tools were employed to manually segment the fibula bone and create a binary mask isolating the bone region (Figure 1A). The mask data were then processed in MATLAB (MathWorks, Natick, MA, USA) version 2024a to extract the length of the centerline (CL, in mm), the central curvature of the fibula bone, through skeletonization using the bwskel function [16,17,18]. This operation reduced the binary mask to a one-pixel-wide skeleton representation of the bone’s medial axis, thereby capturing its central geometry while preserving the overall structure and connectivity.

The fibula bone area (BA) (in mm^2^) was calculated as the number of non-zero pixels in the binary mask multiplied by the voxel area in MATLAB. This calculation was then reverified using Mango’s automatic area calculation, with the final area averaged across seven slices. The fibula cross-sectional bone wall thickness (BT) was computed by averaging the BT measurements from the seven central slices. For each slice, BT was determined by dividing the fibula bone area (BA, in mm^2^) by CL, the centerline between the bone’s outer (D_out_) and inner (D_in_) diameters. This can be expressed mathematically as follows:

(1) $$BT=\frac{1}{7}\sum_{i=1}^{7}\begin{array}{c} \left(\frac{BA}{\;CL\;}\right) \end{array}i$$

where i represents one of the central slices.

**Figure 2:**
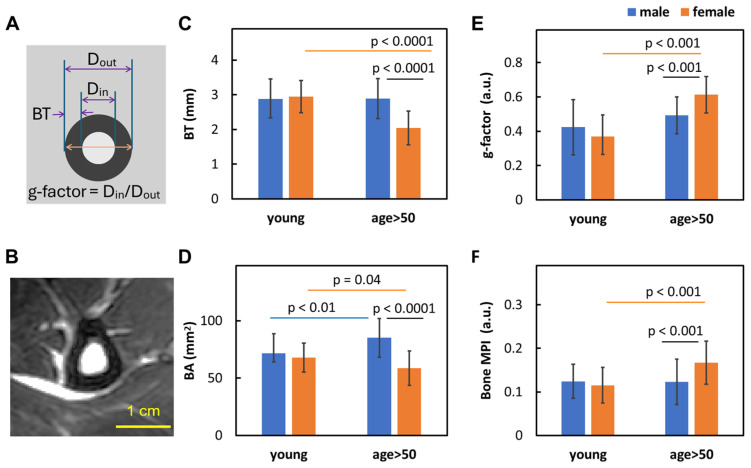
(**A**) A diagram illustrating the ring model used to define fibula bone thickness (BT) and the geometric factor (g-factor). (**B**) A reference MR image. (**C**–**F**) Comparisons of sex differences in bone measurements: (**C**) bone wall thickness (BT), (**D**) cross-sectional bone area (BA), (**E**) g-factor, and (**F**) bone mean pixel intensity (bone MPI) between young (age <= 50, including 17F/13M) and ‘age > 50’ groups (including 26F/51M).

Based on the ring model (Figure 2A), the fibula bone’s geometric factor (g-factor or g-ratio) was defined as the ratio of the bone’s inner (D_in_) to outer (D_out_) diameters and was evaluated by the following:

(2) $$g-factor=\frac{CL-BT}{CL+BT}$$

Bone mean pixel intensity (MPI), an index of BMD loss was measured from the average of the segmented bone area and averaged over the central seven slices. Other fat-related measurements, including bone marrow cross-sectional area (BMA), subcutaneous fat thickness (SFT), and muscle fat infiltration (MFI) and its clustering into normal, mild, and moderate groups by mean and mode pixel intensities, have been reported previously by us [13] and were directly used in correlation with the bone measurements in this study.

The measurement reproducibility, computed by standard deviation divided by average (Δx/$\bar{x}$) was assessed by ten repeated manual ROI segmentations of the same MRI image by the same operator. Then, the resulting measurement variations were used as the input of random noise to evaluate the correlation between the noise-added variables (BT and BA) and demographic factors (age and BMI). The *p*-values of correlations from ten different executions were averaged and compared with the correlations without such noise.

### 2.3. Statistical Analysis

MATLAB’s function *ttest2*, the two-sample *t*-test, was performed to test the null hypothesis that two independent measurements have equal means. The test rejects the null hypothesis at the 5% significance level. MATLAB’s function *corrcoef* was used to evaluate the linear correlation between two diffident sets of measurements, with a *p*-value of < 0.05 presenting statistical significance.

## 3. Results

### 3.1. Size and Shape of Fibula Bone

In cross-sectional MR images, the fibula bone shaft typically appears as an irregular ring with varying bone wall thickness and area (Figure 1). The endosteal contour is largely oval shaped, enclosing bone marrow, while the periosteal contour is largely triangular, with its three sides each attached to a distinct muscle group: flexor hallucis longus/soleus on the posterior side, tibia posterior on the antero-medial side, and peroneus longus/brevis on the antero-lateral side. The cross-sectional shape of the fibula gradually changes along its length; it tends to be larger and rounder at the proximal end (fibular head) and becomes progressively more triangular and sharper toward the distal end. Despite this general topological feature, significant variability in bone size and shape was observed among individuals (Figure 1B). Concave regions, particularly on the antero-medial side, are common, often accompanied by bone wall thinning.

For the cohort of 107 individuals, based on the bone ring model (Figure 2A,B), the outer diameter of the fibula bone (D_out_) averaged 10.9 ± 1.5 mm, with an average bone thickness (BT) of 2.7 ± 0.6 mm. The g-factor ranged from 0.14 to 0.80, with an average of 0.49 ± 0.14. The cross-sectional bone area (BA) averaged 74.3 ± 18.5 mm^2^ compared to the bone marrow area (BMA) 34.5 ± 20.6 mm^2^.

### 3.2. Sex Dependence of Bone Size and Density

As shown in Figure 2C–F, for the younger group (age ≤ 50), there were no significant sex differences in any of the four bone measurements: BT (both 2.9 ± 0.5 mm), BA (M: 71.7 ± 7.7 mm^2^ vs. F: 67.9 ± 12.6 mm^2^), g-factor (M: 0.42 ± 0.16 vs. F: 0.37 ± 0.13), and bone MPI (both 0.12 ± 0.04 mm).

However, for the group aged >50, significant sex differences were observed in all four measurements. Compared to men, women showed lower BT (2.9 ± 0.6 mm vs. 2.0 ± 0.5 mm, Figure 2C) and BA (85.1 ± 16.9 mm^2^ vs. 58.6 ± 15.0 mm^2^, Figure 2D) but a higher g-factor (0.49 ± 0.11 vs. 0.61 ± 0.13, Figure 2E) and bone MPI (0.12 ± 0.05 vs. 0.17 ± 0.05, Figure 2F).

### 3.3. Age Dependence of Bone Size and Density

**Figure 3:**
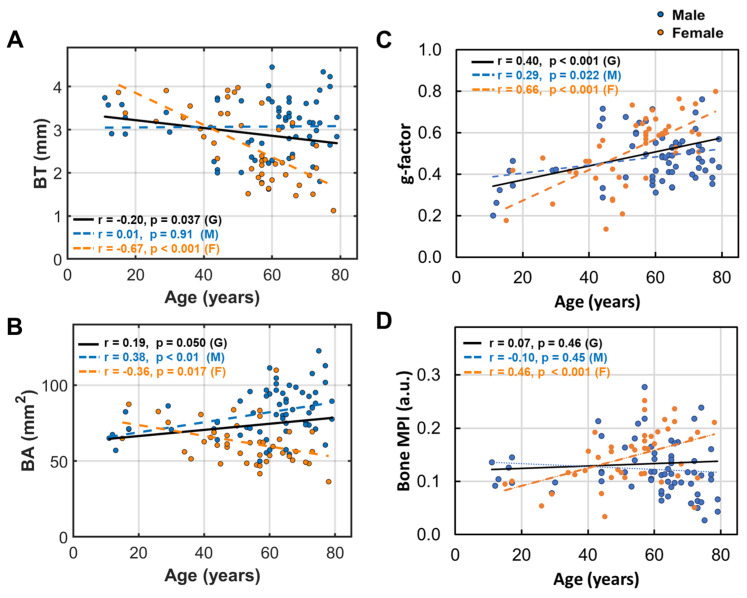
Linear correlation between age and (**A**) bone thickness (BT), (**B**) bone area (BA), (**C**) bone g-factor, and (**D**) bone MPI.

Across the entire age range, fibula bone thickness (BT) decreases with age in women (*p* < 0.01, Figure 3A), while in men, BT remains relatively stable (*p* = 0.94).

Bone area (BA) increases with age in men (*p* < 0.01) but decreases in women (*p* = 0.02, Figure 3B). The fibula bone g-factor increases with age in both groups (men *p* = 0.02, women *p* < 0.01, Figure 3C), with a rate twice as fast in women compared to men. Bone density loss, as indexed by MPI, increases with age, with significance only in women (Figure 3D).

To determine whether the sex difference in bone measurements was influenced by the different age range between men and women, we conducted an analysis where we excluded five subjects from the male group—three from the younger age group (<15 years) and two from the older age group (>78 years). After adjusting the age range to 15–78 years for both men and women, we found that this adjustment did not lead to any significant changes in the [r p] outcomes: [0.08 0.54] for bone thickness (BT), [0.28 0.03] for bone area (BA), and [−0.10 0.44] for bone MPI, as compared to those pre-adjustment [r *p*] values for men (Figure 3).

### 3.4. BMI Dependence of Bone Size and Density

**Figure 4:**
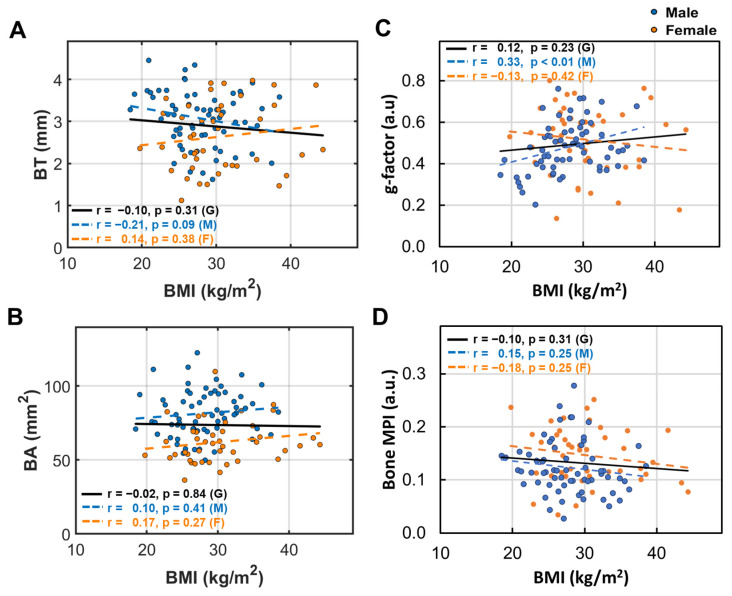
Linear correlation between BMI and (**A**) bone thickness (BT), (**B**) bone area (BA), (**C**) bone g-factor, and (**D**) bone MPI.

Unlike age, BMI does not appear to significantly affect BT, BA, and BL in men and women (Figure 4A,B,D). However, a significant increase in the g-factor was observed in men (*p* < 0.01, Figure 4C).

### 3.5. Bone Marrow’s Correlation with Bone Size and Density

**Figure 5:**
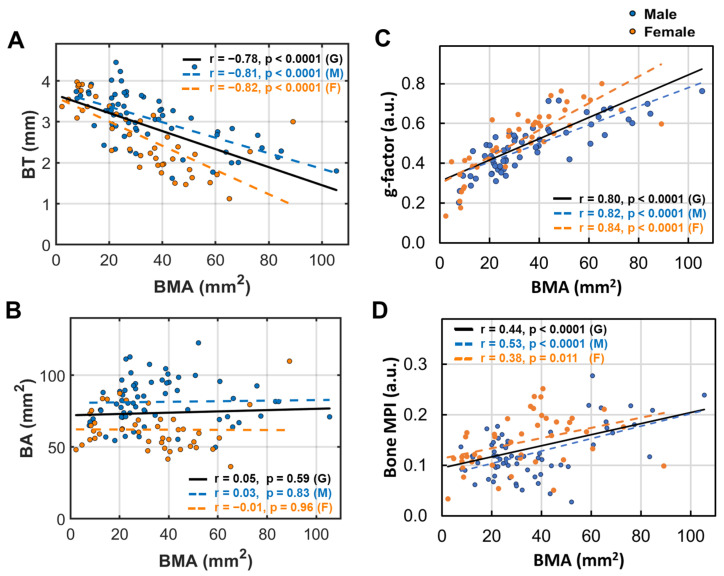
Linear correlation between bone marrow area (BMA) and (**A**) bone thickness (BT), (**B**) bone area (BA), (**C**) bone g-factor, and (**D**) bone MPI.

The fibula bone cross-sectional area (BMA) is significantly correlated with bone thickness (BT) in both men and women (*p* < 0.01, Figure 5A) but not correlated with bone area (BA) in either group (Figure 5B). However, BMA is strongly correlated with the g-factor in both groups (*p* < 0.0001, Figure 5C). Bone density loss increases with BMA in both men (*p* < 0.0001) and women (*p* = 0.011, Figure 5D).

### 3.6. Subcutaneous Fat’s Correlation with Bone Size and Density

**Figure 6:**
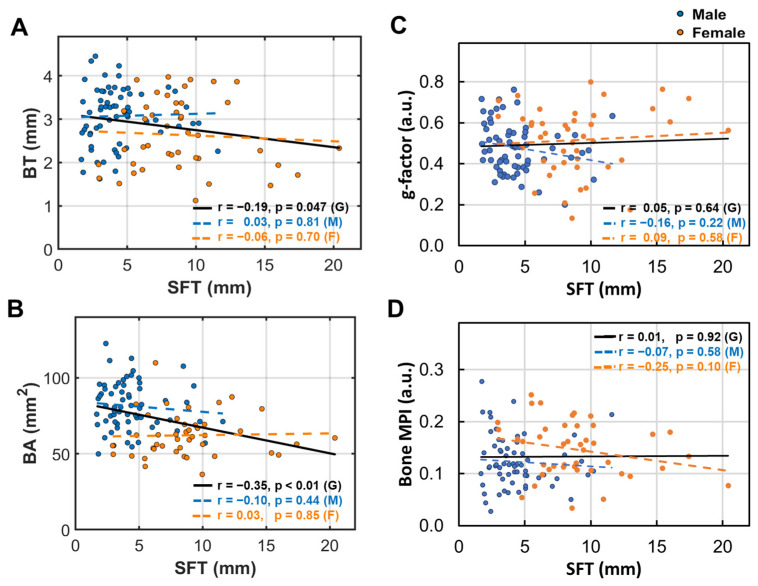
Linear correlation between subcutaneous fat thickness (SFT) and (**A**) bone thickness (BT), (**B**) bone area (BA), (**C**) bone g-factor, and (**D**) bone MPI.

In contrast to BMA, the calf subcutaneous fat thickness (SFT) is not correlated with the BT, BA, g-factor, and bone density loss index in men and women (Figure 6A–D).

### 3.7. Bone Density Decrease in Correlation with Bone Size

**Figure 7:**
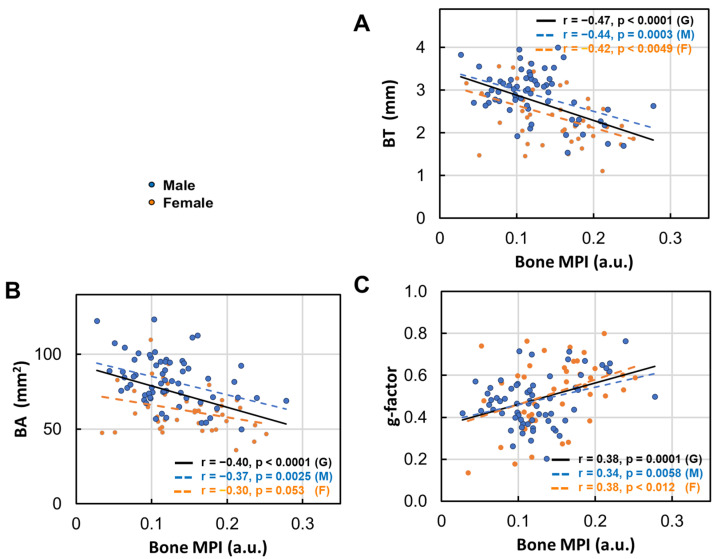
Linear correlation between the bone density decrease index (MPI) and (**A**) bone thickness (BT), (**B**) bone area (BA), and (**C**) g-factor.

Bone density loss tends to increase significantly as bone becomes thinner (*p* < 0.01, Figure 7A), bone area becomes smaller (Figure 7B), and the g-factor becomes larger (Figure 7C), with a higher sensitivity observed in men compared to women.

### 3.8. Muscle Fat Infiltration’s Correlation with Bone Size

**Figure 8:**
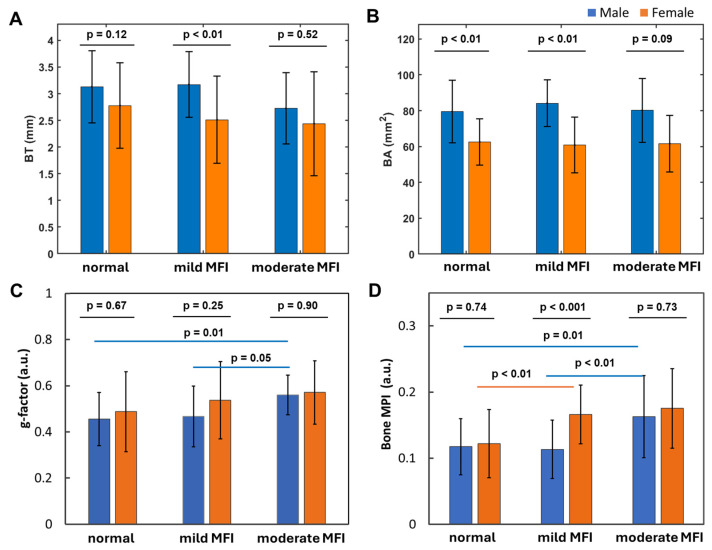
(**A**) Bone thickness (BT), (**B**) bone area (BA), (**C**) bone g-factor, and (**D**) bone MPI at different extents of calf muscle fat infiltration (MFI: normal, mild, and moderate).

Fibular bone thickness (BT) tends to decrease slightly as calf muscle fat infiltration (MFI) increases (Figure 8A), whereas bone area (BA) remains stable in both men and women (Figure 8B). The bone g-factor tends to increase with MFI, with significance observed only in men (Figure 8C). Bone density loss tends to increase with MFI, showing significant differences between normal and mild–moderate MFI in women and between normal–mild and moderate MFI in men (Figure 8D).

Notably, a significant sex difference in BT and bone density was found in the mild MFI group (Figure 8A,D), whereas a significant sex difference in BA was observed in both the normal and mild MFI groups (Figure 8B). However, no sex difference in the g-factor was observed across any of the MFI groups (Figure 8C).

### 3.9. Measurement Variations

Measurement variations from repeated manual ROI segmentations on the same image ranged ± 2.5%, averaging 1.3% for BT and 1.5% for BA, which are approximately 10–20-fold smaller than the corresponding inter-subject measurement variations: 23.9% for BT and 24.9% for BA. No alterations in correlation significance were found when the segmentation variations, plus a 1–3% leeway, were introduced as random noise in BT and BA measurements to assess their correlations with age and BMI.

## 4. Discussion

### 4.1. Key Findings

Musculoskeletal disorders are the leading cause of disability in the U.S., with enormous impacts on quality of life and longevity. Reduced bone mass and density, hallmark features of osteopenia and osteoporosis, are major contributors to increased fractures, falls, and disability in the elderly [19]. This study analyzed MRI scans from 107 patients (64M/43F) aged 11–79, representing diverse ethnicities and a range of BMI. The data reveal several key findings related to fibula bone thinning and bone mineral density (BMD) loss in the lower extremity.

(1) Bone thinning and BMD loss are intricately linked, with a strong correlation that underscores their combined impact on skeletal health (Figure 7).
(2) Women over 50 experience more pronounced bone thinning and BMD loss than their male counterparts in the same age group, highlighting the heightened vulnerability of women to bone loss with age (Figure 3A–D).
(3) In the younger age group (under 50), bone thickness and the bone density index are similar between women and men (Figure 2), suggesting that the significant bone mass reduction and accelerated BMD decrease observed post-menopause may be primarily driven by hormonal changes (Figure 3).
(4) Bone loss (BL), marked by a decrease in bone thickness and density, is closely linked to bone marrow expansion (BME, Figure 5). Both BL and BME accelerate with age yet remain largely unaffected by BMI (Figure 4, and reference [13]), suggesting that bone loss is primarily driven by the aging process rather than the direct effect of BMI increase.
(5) Bone thickness and BMD are largely independent of subcutaneous fat thickness (SFT, Figure 6), although SFT decreases with age in men and increases with BMI in women [13]. Combined with key point #4, this suggests that bone loss is influenced not only by fat accumulation but also by the site of fat deposit with respect to the bone.
(6) Bone loss tends to increase with muscle fat infiltration (MFI) (Figure 8). However, the detailed aspects of this bone–MFI relation are sex dependent, with bone area (BA) being more sensitive to MFI compared to bone thickness (BT). This likely reflects the anatomic relationship between bone and muscle.

### 4.2. Fat Distribution Matters

Our findings reveal that regional adiposity plays a crucial role in bone loss, with effects depending on the proximity of fat depots to the bone. Notably, bone marrow fat appears to have the most profound impact on bone lossfollowed by fat infiltrating muscle tissue. In contrast, subcutaneous fat and more distal body fat (as reflected in the bone–BMI relationship) have much less influence. This aligns with the established link between increased bone marrow fat and osteoporosis [19,20,21,22].

Our finding of bone thinning in correlation with bone marrow expansion suggests that bone resorption by osteoclasts dominates on the endosteal surface, while bone formation by osteoblasts dominates on the periosteal surface. Additionally, the correlated increase in MPI in both muscle and bone (Figure 8), along with their parallel rise with aging (Figure 3D and reference [13]), suggests that fat infiltration may occur in bone similar to skeletal muscle.

Skeletal muscle is the body’s largest organ by mass and plays a crucial role in regulating glucose metabolism. When excessive fat accumulates in muscle, it impairs the muscle’s ability to process glucose, increasing the risk of insulin resistance and associated health conditions such as obesity, heart failure, stroke, sarcopenia, chronic kidney disease, type 2 diabetes, and osteoporosis. By linking MFI, BME, and bone loss, this and our previous study [13] highlight a potential avenue for dual-targeted therapies that support both muscle and bone health.

In contrast to aging and BME, our data also show that bone loss is not correlated with BMI, which contributes to the growing body of evidence that challenges the long-held belief that obesity is protective against the development of osteoporosis [20,23]. While we agree that mechanical loading has positive effects on bone health, body overweight may not be sufficient to offset other negative effects of obesity, such as low-grade systematic inflammation, which is detrimental to bone health (see below). Therefore, the effects of obesity on bone loss should also be examined in light of fat distribution. While increased subcutaneous fat may not pose a significant concern, higher levels of visceral fat within muscle and bone could be a risk factor.

Adipocytes, particularly those in bone marrow and muscle, have been recognized as important players in the inflammatory response, secreting adipokines (e.g., leptin and adiponectin) and pro-inflammatory cytokines, which can stimulate or exacerbate bone resorption and loss. Additionally, the skeletal system contains a series of sophisticated cellular lineages arising from the mesenchymal stem cells (MSCs) and hematopoietic stem cells (HSCs) that determine the formation of bone vs. bone marrow [20]. Furthermore, bone marrow serves as a niche housing for a variety of immune cells, including macrophages, dendritic cells, T-cells, and B-cells, all of which are involved in inflammatory processes [24]. These immune cells can produce pro-inflammatory cytokines, such as TNF-α, IL-1, IL-6, and IL-17, which can act on nearby osteoblasts, osteoclasts, and other bone-residing cells, contributing to bone resorption and loss [24,25,26].

### 4.3. Aging Effects

Our findings indicate that bone loss becomes pronounced after age 50, especially in women. This aging effect may be due to several interconnected factors: hormonal changes, inflammation, metabolic shifts, and structural changes in bone and surrounding soft tissues [27]. Especially, during aging, senescent cells (SnCs) tend to accumulate in the bone, triggering chronic inflammation by releasing senescence-associated secretory phenotype (SASP) factors [28]. Aging is also associated with hypometabolism, which is manifested by declining hormonal secretion and a reduced drive for physical activity. These changes can shift bone remodeling toward increased resorption and reduced formation [27,28].

It is known that aging-derived chronic low-grade inflammation, or ‘inflammaging,’ triggers the release of pro-inflammatory cytokines (e.g., TNF-α, IL-6), which in turn enhance osteoclast activity and accelerate bone resorption [28,29,30]. Additionally, with aging, both calcium absorption in the gut and vitamin D synthesis in the skin tend to decrease, impairing bone mineralization and ultimately contributing to net bone loss [31,32].

### 4.4. Sex Matters

Figure 2 and Figure 3 clearly show that aging-dependent bone loss tends to affect women significantly more than men, particularly those over age 50, including post-menopausal women and those transitioning into menopause (note: in the U.S., the average age for menopause is around 51). Compared to men, women over age 50 exhibit 29% lower bone thickness (Figure 2A), 31% lower bone area (Figure 2D), a 24% larger g-factor (Figure 2E), and a 36% larger bone loss index (Figure 2F). The primary factor contributing to this sex difference may be the sharp decline in estrogen levels that occurs during menopause [33]. In contrast, men typically experience a more gradual decline in bone density with aging, likely due to their slower rate of testosterone decline. Both estrogen and testosterone are known to inhibit osteoclast activity for bone resorption and stimulate osteoblast activity for bone formation [34,35,36].

Another interesting observation is that women over 50 experience both bone mass loss and bone density loss, while men over 50 experience only bone density loss without a decrease in BT and BA (Figure 2C,D,F). This suggests that the osteal structure may be compromised in women but not so much in men, a finding consistent with the higher prevalence of bone density loss and osteoporosis in women, affecting 10% of women compared to 2% of men over the age of 50 in the U.S. [37,38]. In addition to hormonal differences, other sex disparities that disproportionately affect women’s bone health also include less physical strength and less participation in outdoor activities, among others [39,40].

### 4.5. g-Factor

In this study, we introduce the g-factor, or g-ratio, defined as the ratio of a bone’s inner-to-outer diameters (Figure 2A), which reflects the thickness of the bone wall relative to the enclosed marrow cross-sectional diameter. This concept is analogous to the g-ratio used to describe axonal myelination in the CNS, where a higher g-ratio indicates a thinner myelin sheath relative to the axon diameter [41]. In this sense, bone thinning is analogous to demyelination in three aspects. (1) Both conditions tend to worse with aging. (2) Both conditions are implicated with inflammation, with the participation of neighboring bone marrow (i.e., skull marrow in CNS demyelination) [42,43,44,45]. (3) Both are more prevalent in women compared to men, as indicated in cases of osteoporosis and multiple sclerosis.

For a constant bone wall thickness, an increased g-ratio reflects either absolute thinning of the bone wall (decreased BT), relative thinning, or both. Thus, the g-ratio serves as an important index of bone thinning, complementing BT. This unique feature of the g-ratio is evident in Figure 8, where it effectively distinguishes the impact of varying MFI severities (Figure 8C), whereas BT does not (Figure 8A). Similarly, in Figure 4, while BT shows no clear pattern with BMI (Figure 4A), the g-ratio detects BMI-dependent changes, revealing a relative bone thinning with an increase in BMI in men (Figure 4C). In summary, the g-ratio’s role in bone loss is as critical as its role in describing demyelination in the CNS.

### 4.6. Other Remarks

So far, we have examined bone loss from a pathological perspective. However, it is important to recognize that regional and temporal bone loss can be a natural process in the context of normal bone remodeling. Bone not only provides structural support for movement but also serves as a reservoir for essential minerals, such as calcium, phosphate, and magnesium, which may be released when more critical cellular functions require them. Deficiencies in these minerals can have profound effects on muscle and brain health due to their crucial role in energy metabolism, control of synaptic activity, and memory formation [46]. Thus, regional cavities and pores in bone might be a result of the mobilization of these minerals to meet the needs of other more vital cells during times of mineral deficiency in circulation. Therefore, to improve personal bone health, it is important to consume a balanced diet rich in essential minerals, participate in regular physical activity (especially outdoor exercise with sun exposure), reduce sedentary behavior (such as prolonged sitting and screen time), and if necessary, take mineral supplementations that are tailored to individual needs in composition and dosage.

Another issue worth pondering is the relationship between bone loss and bone marrow expansion. Is bone marrow expansion a passive aging process, or is it a necessary adaptation to compensate for dysfunction in other tissues? Bone marrow resides in a physically protected space where blood vessels are resilient, ensuring circulation even when other tissues are compromised or dysfunctional. Intraosseous (IO) access provides a fast and reliable route for medication and infusion delivery, especially when standard venous access is difficult or could be delayed [47]. In this context, it is possible that bone marrow expansion (BME) serves as a natural part of the aging process, helping to ensure critical circulation by bypassing dysfunctional regions in neighboring soft tissues that are compromised or at an increased risk of damage with aging. Alternatively, BME may represent a space-filling effect, serving as storage for long-term fuel following bone resorption at the endosteal surface. Both BME and bone loss are more pronounced in individuals over age 50 compared to younger individuals (Figure 2 of this study and Figure 4A of reference [13]).

It is worth noting that while techniques such as DXA (Dual-energy X-ray Absorptiometry) and CT (Computed Tomography) are widely used for assessing bone mineral density and structural properties [48,49], T2-weighted MRI offers unique advantages in distinguishing between bone, marrow, and muscle due to its ability to differentiate soft tissue components based on water content. Unlike DXA and CT, which focus primarily on bone density, T2w MRI provides more detailed and specific tissue contrast without the need for ionizing radiation, making it particularly useful for studying bone marrow and muscle in addition to bone. These features make T2w MRI a preferable choice for our study’s objectives.

### 4.7. Limitations

This study highlighted the interplay of bone, muscle, and marrow by examining bone loss through four complementary measurements, BT, BA, g-factor, and bone MPI, which encompass indexes of both bone mass and density. Aging, sex, and fat distribution all matter to bone health. However, the underlying molecular pathways and mechanisms of bone loss remain to be explored. Future research should focus on (1) unambiguously identifying the molecular sources responsible for the intraosseous 1H MRI signals, (2) integrating MRI with other metabolic imaging techniques, like magnetization transfer (MT), bone marrow 1H MRS, and skeletal muscle 1H/31P MRS [50,51,52], and (3) exploring in depth the effects of minerals (supplements and dietary) on bone and muscle health [53,54,55]. Another limitation of this study is the restricted ROI coverage, focusing only on the compact bone of the fibular bone shaft, as constrained by the size of our 7T RF coil for calf use and described in our previous study [13]. Future efforts using RF coils with expanded coverage for the fibula’s proximal trabecular bone, as well as the tibia, could further enhance our understanding of bone loss characteristics. Additionally, we did not explore the connection between fibula bone shape and muscle morphology, as well as their variations among individuals, due to the limited space and scope of this study. Further research is needed to investigate issues such as why some individuals have fibulas with pitted and uneven periosteal surfaces, whether the formation of runway-like bone outgrowth in certain individuals reflects long-term changes of the attached muscles, and how genetic factors and mechanical loading—such as repeated stress and activities (e.g., walking habits and sports)—may (re)shape the fibula. Furthermore, it should be noted that such morphological characteristics and dimensions of the fibula are valuable for evaluating the suitability of specific bone areas for use as donor sites in dental reconstructions involving implants [56].

In conclusion, our study highlights the utility of 7T MRI in assessing fibular bone thickness and density, yielding significant insights into bone loss. We found that in women over 50, bone loss is characterized by a decrease in both bone mass and density, whereas men in the same age group primarily experience bone density loss. This sex difference in bone structure may contribute to the higher prevalence of osteoporosis in women. Furthermore, bone marrow expansion (BME) and muscle fat infiltration (MFI) were closely associated with bone loss, with BME potentially acting as a response to bone thinning and potentially contributing to further bone loss through inflammation in the marrow. Our analysis also suggests that unlike age, sex, BME, and MFI, body mass index (BMI) and subcutaneous fat thickness (SFT) do not appear to be critical factors in bone thinning and density loss. Importantly, we introduce the g-factor (inner-to-outer bone diameter ratio) as a novel marker for bone thinning, underscoring its potential as a key parameter in assessing bone health. Finally, to prevent and manage bone loss, particularly in women over 50, a multifaceted approach is crucial. This should include regular weight-bearing and resistance exercises to enhance bone strength, adequate intake of calcium and vitamin D to support bone mineralization, and, where appropriate, pharmacological treatments. Additionally, addressing modifiable risk factors such as physical inactivity, poor diet, and chronic low-grade inflammation can further help reduce the risk of bone deterioration and fractures. By combining lifestyle changes, nutritional support, and medical treatments, the impact of age-related bone loss can be mitigated, and this would empower individuals to maintain stronger, healthier bones into their later years.

## Data Availability

Data in this study are available upon request to the corresponding authors.
